# Beyond the Cholesterol-Lowering Effect of Soy Protein: A Review of the Effects of Dietary Soy and Its Constituents on Risk Factors for Cardiovascular Disease

**DOI:** 10.3390/nu9040324

**Published:** 2017-03-24

**Authors:** D. Dan Ramdath, Emily M. T. Padhi, Sidra Sarfaraz, Simone Renwick, Alison M. Duncan

**Affiliations:** 1 Guelph Research and Development Centre, Agriculture and Agri-Food Canada, Guelph, ON N1G 5C9, Canada; epadhi@gmail.com (E.M.T.P.); sidra9518@gmail.com (S.S.); srenwick@mail.uoguelph.ca (S.R.); 2 Department of Human Health and Nutritional Sciences, University of Guelph, Guelph, ON N1G 2E1, Canada; amduncan@uoguelph.ca

**Keywords:** cardiovascular disease, cholesterol, functional foods, isoflavones, lipids, obesity, Dietary soy, soy protein

## Abstract

The hypocholesterolemic effect of soy is well-documented and this has led to the regulatory approval of a health claim relating soy protein to a reduced risk of cardiovascular disease (CVD). However, soybeans contain additional components, such as isoflavones, lecithins, saponins and fiber that may improve cardiovascular health through independent mechanisms. This review summarizes the evidence on the cardiovascular benefits of non-protein soy components in relation to known CVD risk factors such as hypertension, hyperglycemia, inflammation, and obesity beyond cholesterol lowering. Overall, the available evidence suggests non-protein soy constituents improve markers of cardiovascular health; however, additional carefully designed studies are required to independently elucidate these effects. Further, work is also needed to clarify the role of isoflavone-metabolizing phenotype and gut microbiota composition on biological effect.

## 1. Introduction

Cardiovascular disease (CVD) describes a collection of disorders affecting the vasculature of the heart, brain and peripheral tissue and is the leading cause of death globally [1]. Atherosclerosis is the underlying cause of coronary heart disease (CHD), the most common form of CVD, and is thought to be initiated through an inflammatory response by the vascular endothelium following injury [2]. The origin of these endothelial lesions is unclear, but implicated factors include: chronic elevations in blood pressure [3]; prolonged hyperglycemia and the resulting formation of advanced glycation end products [4]; elevated LDL cholesterol (LDL-C), particularly molecules that have undergone oxidized modification [2]; and oxidative stress and inflammation [5]. Consequently, major CVD risk factors include hypertension, the presence of type 2 diabetes, dyslipidemia, obesity (body mass index (BMI) >30), and inflammation [1]. Dietary modification lowers CVD risk by attenuating associated risk factors, and in particular, legumes are emphasized as part of a cardioprotective diet because increased consumption is associated with improved weight management and glycemic control, reduced blood pressure, and an improved plasma lipid profile [6].

Soybeans (*Glycine max*) are widely cultivated for their lipid content, and indeed are the top oilseed produced worldwide [7]. In addition, soybeans are recognized as a valuable source of nutrients as they contain high-quality protein (~40%); polyunsaturated fatty acids (18%); carbohydrates (primarily sucrose, stachyose, and raffinose); and dietary fibers [8]. Soy foods have been part of the human diet for millennia, but more recently considerable attention has been given to the associated health benefits of soy, particularly reduction of CVD risk via the lowering of LDL-C. Much of the focus on soy has been directed toward the hypocholesterolemic properties of bioactive peptides in soy protein, which exert their effects primarily through mechanisms involving the LDL-C receptor (LDLR), and bile acid regulation [9,10]. These findings are supported by several meta-analyses [11,12,13,14,15,16,17,18,19,20,21] and have culminated in a soy health claim relating 25 g soy protein with a reduced risk of CHD in the United States [22] and Canada [23], but not Europe [24]. However, other constituents in soy have been shown to confer many health benefits, including reduction of CVD risk, and these are worthy of further examination.

Soybeans are a significant source of phytochemicals such as isoflavones, phytosterols and lecithins, as well as soluble fibers, saponins and polysaccharides, which may act collectively or through independent mechanisms to confer unique health benefits [25,26,27]. For example, soy lecithins and saponins have a role in lipid metabolism; phytosterols and linoleic acid produce hypocholesterolemic effects [25]; and soy fibers have been shown to promote weight loss [28]. Further, the health benefits of soy protein appear to reach beyond its putative LDL-C lowering effect by offering protection against renal dysfunction [29], oxidative stress [30], and by improving markers of endothelial function [31]. Finally, the health benefits of isoflavones, of which soybeans are the single greatest dietary source, have also been extensively examined. They represent a class of phytoestrogens belonging to the flavonoid family, and there are three primary isoflavones (daidzein, genistein, glycitein) which are characterized by their general di-phenolic structure resembling mammalian estrogen [32]. The structural similarity between soy isoflavones and estradiol suggests that isoflavones may elicit estrogenic effects. Although the affinity for the estrogen receptor by soy isoflavones is 100–1000 times less than mammalian estrogen, isoflavone concentrations can appear in plasma >1000-fold greater than that of endogenous estrogen, thus lending support to the suggestion that isoflavones can exert significant physiological effects [33].

The cholesterol-lowering effect of soy protein has generated much interest in the past and has been extensively studied, but other components in soy appear to confer significant cardiovascular health benefits despite receiving less attention over the years. This review serves to examine and expand on the health effects of soy and soy constituents beyond cholesterol reduction. Herein, we provide an up-to-date summary of the epidemiological and clinical evidence associating dietary soy, and other soy-derived components, with reduced CVD risk. This review will examine the cardio-protective effects of dietary soy in the context of major disease outcomes such as hypertension, hyperglycemia, dyslipidemia, inflammation, and obesity.

## 2. Epidemiological Studies

Current epidemiological findings show an inverse association between consuming whole soy foods/products and CVD risk. For example, Shimazu et al. (2007) studied Japanese dietary patterns and found that increased soybean intake (up to 101 g/day) was associated with lower CVD mortality [34]. However, the recent Takayama study, which examined dietary intakes of soy and natto (fermented soy beans) and CVD mortality among Japanese adults, found that there was a significant decrease in mortality from stroke at the highest quartiles of total soy protein and natto intake [35]. Despite this finding, the authors conclude that except for natto, there were no significant associations between CVD related mortality and intakes of total soy protein, total soy isoflavone, and soy protein or soy isoflavones from soy foods [35]. It appears that different soy foods may vary in their biological efficacy and protective effects. In this regard, it is worthwhile to examine the role of soy isoflavones, given that their estrogenicity may protect against the sharp rise in CVD incidence after menopause, when endogenous estrogen concentrations are depleted [15]. Although plausible mechanistic evidence supports the cardio-protective effects of soy isoflavones, there is discrepancy in the literature regarding the health effects of isoflavones extracts that are consumed in isolation of other soybean components, and this has created disagreements about their health benefits.

Observational studies among East Asian populations consistently find that chronic disease incidence is lowered with the intake of isoflavone-containing whole soy and traditional soy-based foods [36]. In contrast, studies performed with Western populations frequently utilize various combinations of isolates to evaluate the health effects of soy, and have failed to consistently demonstrate health benefits [36]. Furthermore, there is emerging evidence on the diverging biological effects among individuals capable of metabolizing the soy isoflavone daidzein to the more bioactive metabolite equol, which suggests a complex interplay between gut microbiota composition and host benefit, perhaps accounting for some of the conflicting results in the literature [37]. For example, in exploring the effect of soy on CVD risk factors, it was found that blood pressure, plasma triglycerides (TG) and C-reactive protein (CRP) concentrations were significantly lowered among equol- and *O*-desmethylangolensin (ODMA)-producing pre-hypertensive, postmenopausal Chinese women, compared to non-producers [38]. In addition, racial differences in the ability to convert daidzein into equol have been reported. For example, 30%–40% of Western populations are estimated to be equol producers while greater proportions have been observed in Japanese, Chinese, and Korean populations, which are purportedly linked to differences in genetics, gut microbiome composition, and perhaps diet [39]. Interestingly, background diet did not influence isoflavone bioavailability in Chinese Asian adults who were acclimatized to a French Western dietary pattern [40].

Other health benefits observed among soybean consumers include lower BMI and mortality due to CHD [41], while lower circulating inflammatory markers are observed among female soy consumers [42]. Soy consumption has also been associated with the lowering of blood pressure. In the US, a pooled analysis of three longitudinal cohort studies (Nurses’ Health Study, Nurses’ Health Study II and Health Professionals Follow-up Study) found that consuming ≥4 servings/week of broccoli, carrots, and tofu/soybeans was associated with a lower risk of hypertension compared to consuming <1 serving/month [43]. However, the Tehran Lipid and Glucose Study, which involved 1546 normotensive adults, did not find an association between incident hypertension and dietary phytochemical intake, which provided an indirect estimate of soybean consumption [44].

Soy intake may also lower CVD risk by mitigating risk factors for type 2 diabetes (T2D), although this protective effect appears to be limited to populations living in East Asia. In the Saku Study, which stratified participants by sex and BMI, Japanese men with a higher BMI (>23.6 kg/m^2^) who consumed soybean products ≥4 servings/week experienced lower fasting and postprandial blood glucose concentrations, and lower T2D incidence compared to those consuming <1 serving/week [45]. Additionally, higher intake of soy products, daidzein and/or genistein was associated with lower T2D risk among overweight Japanese women [46] and adult Chinese Singaporeans [47]. By contrast, intake of soy foods (tofu/soy milk) among US adults does not appear to lower T2D risk, although an inverse relationship between soy isoflavone intake and T2D risk was identified in one study [48]. Similarly, a multi-ethnic cohort study conducted in Hawaii did not find an association between soy food intake and diabetes risk among Caucasian, Japanese American, and Native Hawaiian adults [49]. More recently, a meta-analysis was unable to establish a relationship between soy intake and risk of stroke or CHD, although the authors reason that this may be attributed to the limited pool of case-control and cohort studies currently available [50]. Taken together, population-based studies suggest soy intake mitigates CVD risk factors, but it is unclear whether Western populations gain similar health benefits observed among populations living in East Asia. The results of observational studies conducted in Western populations often lack rigor and are controversial, quite possibly because the active components of soy-based interventions are not adequately characterized. Additionally, it is possible that there are coexisting dietary and environmental factors in East Asian cultures that confound the outcome of these studies.

## 3. Interventional Studies Involving Soy

### 3.1. Blood Pressure Lowering Effect of Soy

Hypertension is defined as systolic blood pressure (SBP) >140 mm·Hg and/or diastolic blood pressure (DBP) >90 mm·Hg [51]. Primary hypertension accounts for 90% of all cases and although the underlying cause is unclear, the major contributing factors include diet, smoking, stress, obesity [51], and possibly genetics [52]. Hypertension increases the risk of vascular injury through pro-inflammatory mechanisms, thereby increasing CVD risk. Further, angiotensin II, a potent vasoconstrictor, is elevated during hypertension and can increase the activity of the free radical-generating lipoxygenase enzyme in smooth muscle cells, which contributes to the formation of oxidized LDL-C (oxLDL), superoxide anion, and hydroxyl radicals [2]. Soy and some of its constituents have been shown to reduce risk for hypertension through effects on vasodilation and inhibition of a key enzyme involved in the regulation of blood pressure.

Isoflavones have been shown to mitigate hypertension by targeting mechanisms involving vasodilation; in particular, interaction with the estrogen-response element of genes related to endothelial nitric oxide (NO) synthase increases endogenous NO production, which improves brachial artery flow [53]. In postmenopausal women, six months of isoflavone supplementation was shown to improve endothelial vasodilation and resulted in a significant reduction in cellular adhesion molecules such as Intercellular Adhesion Molecule 1, Vascular Cell Adhesion Protein 1, and E-selectin [54]. In support of these findings, animal studies have shown that soy isoflavones increase renal blood flow, sodium excretion, and interact with estrogen receptors to inhibit angiotensin converting enzyme activity in the renin-angiotensin-aldosterone system [55]. However, clinical evidence supporting a role for isoflavones in hypertension management remains controversial. A meta-analysis of 14 randomized controlled trials (RCTs) found that daily intake of isoflavone extracts (25–375 mg) over a period of 2–24 weeks significantly decreased SBP (−1.92 mm·Hg, 95% CI: (−3.45 to −0.39 mm·Hg)), but not DBP, in normotensive adults [56]. Another meta-analysis found that daily intake of soy isoflavones (65–153 mg) for 1–12 months significantly lowered blood pressure (SBP: −5.94, 95% CI, (−10.55, −1.34 mm·Hg); DBP: −3.35, 95% CI, (−6.52, −0.19 mm·Hg)) in hypertensive, but not normotensive, adults [57]. This suggests that the hypotensive effect of isoflavones is best achieved in persons with established hypertension. Recently, a meta-analysis of 71 trials investigating the effect of phytoestrogen supplementation on arterial hypertension concluded that reductions in SBP and DBP were not significant [58].

Several studies suggest that the effect of isoflavones on endothelial function may be related to individual capacity to metabolize daidzein into equol [59]. Recently, a double-blind crossover study demonstrated significant improvements in arterial stiffness, blood pressure, and endothelial function with the consumption of purified equol supplements, but only among equol-producing men [60]. Another study showed that soy nuts, which provide a source of both soy protein and isoflavones, attenuated SBP in both healthy women and those with metabolic syndrome (MetS), but DBP was significantly decreased only in participants identified as equol-producers [61]. In equol-producing women with prehypertension or untreated hypertension, neither whole soy nor purified daidzein caused significant changes in 24-h ambulatory blood pressure after six months [62].

Apart from isoflavones, other components of soy have been shown to possess hypotensive properties. Soy pulp, which contains oligopeptides and high amounts of fiber, has exhibited anti-angiotensin-converting enzyme activity in vitro, providing mechanistic evidence for a hypotensive effect [63]. Previously published systematic reviews have indicated difficulty in ascertaining the effect of soy protein on blood pressure, citing a lack of studies that focus solely on the protein component of soy as the active agent [31,64]. Soy protein consumed in combination with isoflavones appears to lower SBP in pre-diabetic, postmenopausal, hypertensive women when compared to milk protein [65,66], and a recent meta-analysis showed that soy protein lowers DBP in patients with T2D and MetS [67]. In another study, nut consumption was associated with lower SBP and DBP in adults without T2D; however, subgroup analysis revealed that soy nuts alone did not reduce blood pressure [68]. Further, a novel bread fortified with soybean flour did not improve blood pressure in women with T2D [69], and soy lecithin with and without isoflavone-rich soy protein isolate did not ameliorate brachial artery flow-mediated dilation, although improvements in the plasma lipids profile were observed [70]. The amino acid composition of dietary soy is another interesting feature that may help explain its associated hypotensive effects. Soy foods and legumes in general, are rich in arginine which is a precursor to NO in the l-arginine-nitric oxide pathway [71]. It is thought that arginine aids in regulation of blood pressure through increased production and improved NO bioavailability in the vascular endothelium [72]. Two meta-analyses have concluded that supplementation with l-arginine significantly improves blood pressure and endothelial function in adults [72,73]. Additionally, soy foods have increased arginine content relative to lysine, which may impact the hypotensive activity of soy containing foods. Both amino acids compete for the same transporter in the intestinal lumen, so increased lysine relative to arginine could limit uptake of the latter, and thus affect its bio-conversion and consequent downstream hypotensive effects [74]. Table 1 shows the arginine and lysine contents of soy protein isolate relative to other protein sources.

Overall, although soy isoflavones seem to attenuate blood pressure, this effect is more likely to occur in hypertensive or equol-producing individuals. The independent and/or combined hypotensive effects of other soy components such as soy protein and its amino acid composition, fiber, lecithins, and saponins require further studies as the current body of evidence is limited by that number of available RCTs, but currently does not suggest any significant hypotensive effects.

### 3.2. Blood Glucose Lowering Effect of Soy

Hyperglycemia is defined as chronically elevated fasting blood glucose (FBG) >6.1 mmol/L that typically results from abnormal glucose metabolism and impaired insulin sensitivity [80]. Greater CVD incidence among diabetic patients suggests that hyperglycemia is associated with an increased CVD risk, perhaps by exacerbating atherosclerotic lesions through the generation of advanced glycation products [4,81]. Murine models of T2D have shown that supplementation with soy isoflavones lead to favorable effects, such as reduced islet β-cell loss and increased antioxidant enzyme activity [82,83,84]. In particular, genistein has been shown to possess antioxidant activity and inhibit tyrosine kinase, which collectively may alter insulin secretion. However, quite independently, genistein appears to directly affect β-cell proliferation, glucose-stimulated insulin secretion and confers protection against apoptosis through mechanisms that involve cyclic AMP/Protein Kinase A (cAMP/PKA) signaling as well as epigenetic regulation of gene expression at physiologically relevant doses [85]. Phenolic-rich extracts from soybeans also appear to inhibit α-amylase and α-glucosidase enzymes in vitro, potentially aiding in the regulation of postprandial blood glucose [86]. These studies demonstrate plausible mechanistic evidence supporting the role of soy isoflavones in improving glycemic control, and are supported by human trials that show soy isoflavones can significantly alter glucose homeostasis. For example, 120 postmenopausal Caucasian women with MetS experienced significant improvements in FBG, insulin, and insulin sensitivity after consuming 54 mg of purified genistein daily for one year [87]. Chinese women given a calcium supplement containing 40 or 80 mg soy isoflavones experienced significant reductions in FBG over the course of one year compared to a group given a calcium supplement alone [88]. In addition, FBG, insulin, and insulin resistance decreased in postmenopausal women at risk for osteoporosis following long-term supplementation with genistein extracts [89]. However, other markers of glycemic control, such as 2-h postprandial glucose and HbA1c did not improve when Chinese women consumed soy protein with or without intact isoflavones [90], or when 50 mg of daidzein or genistein supplements were ingested daily for 24 weeks [91]. A meta-analysis that examined the effect of soy isoflavones supplementation in peri- and postmenopausal non-Asian women found significant treatment effects, including reduced circulating insulin (−1.37 µIU/mL, 95% CI: (−1.92 to −0.81 µIU/mL)) and insulin resistance as measured by the Homeostatic Model Assessment for Insulin Resistance (HOMA-IR) (−0.39, 95% CI: (−0.65 to −0.14)) but not FBG [92]. A more recent meta-analysis concluded that isoflavones, particularly genistein, significantly lowered FBG (−0.22 mmol/L, 95% CI (−0.38 to −0.07 mmol/L) and insulin resistance (HOMA-IR, −0.52, 95% CI: (−0.76 to −0.28) in postmenopausal women, although substantial heterogeneity between studies was noted [93]. Another recent meta-analysis of individuals with T2D and MetS showed significant reductions in FBG and insulin concentrations, compared to placebo, when soy protein was consumed for ≥6 months [94].

Closer examination of the available studies suggests that improvements in glycemic control are achieved more frequently with soy foods rather than isolates. For example, a meta-analysis of 24 randomized controlled trials found no significant improvements in FBG, insulin, or HbA1c with the consumption of soy protein with or without isoflavones; however, a subgroup analysis showed that whole soy, but not purified isoflavones significantly reduced hyperglycemia [95]. Among older women with MetS, significant reductions in FBG, insulin, and HOMA-IR were experienced with soy nuts but not textured soy protein [96]. A similar finding was made in a study that tested the effect of soy nuts or soy protein isolate on blood glucose response in postmenopausal women with MetS [97]. In persons with T2D, blood glucose and insulin responses were lowered in those consuming a nutrition bar made from soybeans compared to an isocaloric cookie control [98], and whole soy powder significantly reduced the glucose, but not insulin, response compared to white rice [99].

The diverging effect of whole soy foods compared to soy protein isolates suggests that other components (e.g., fiber, saponins, polysaccharides, phytosterols) may account for the hypoglycemic effect of soy [95]. Indeed, soluble fibers extracted from soy hulls have *in vitro* binding capacity and physicochemical properties (solubility, viscosity, water-holding capacity) similar to oat β-glucan, a component of oat fiber with well-documented cholesterol and glucose-lowering properties [100]. In a randomized crossover study, soluble polysaccharides extracted from soy did not alter postprandial blood glucose response in healthy young males; however, an inverse relationship was found between product viscosity (but not fiber concentration), glucose area under the curve (AUC), and glycemic index [101]. Currently, a lack of human studies examining the hypoglycemic effect of soy components other than isoflavones and protein makes it difficult to draw conclusions on the effects of these components. Additional studies are therefore required in order to better clarify the role of these bioactive agents in potentiating the hypoglycemic effect of soy. A summary of findings from randomized control trials based on the impact of soy on hyperglycemia is presented in Table 2.

### 3.3. Non-Protein Effects of Soy on Blood Lipids

Studies examining the relationship between soy and dyslipidemia, defined as elevated plasma LDL-C and TG concentrations and often accompanied by low HDL-C [102], have highlighted differences in the efficacy between soy isoflavones and protein in reducing LDL-C. In many human trials, a hypolipidemic effect has been demonstrated with soy isoflavones; however, this effect appears to be inconsistent so there is wide disagreement in the literature. Isoflavones are believed to exert their hypolipidemic effects by binding with estrogen receptors when circulating estrogen is low, after which they translocate to the nucleus, bind to DNA sequences near the promoter region of target genes, and induce DNA transcription [103]. Through this mechanism, isoflavones could serve as ligands for lipid-regulating proteins such as the peroxisome proliferator activated receptors (PPARs), the liver X receptor, and the farnesoid X receptor, which would lower hepatic lipid synthesis, bile acid synthesis, and cholesterol reabsorption [104]. As such, there is a mechanism by which soy isoflavones could theoretically modulate circulating cholesterol levels. However, isoflavone supplements taken by postmenopausal women for 12 weeks did not alter the expression of genes associated with the LDLR and scavenger receptor CD36, both of which are important in regulating plasma LDL-C concentrations [105]. Further, no significant changes in body fat and visceral adipose tissue were detected; in fact, a potentially deleterious significant increase in LDL-C was observed [105]. This finding agrees with a meta-analysis which found that an average of 70 mg/day purified isoflavone aglycone extracts (27–132 mg/day), consumed independently of soy protein, did not significantly lower total or LDL-C in normocholesterolemic menopausal women [18]. Another meta-analysis found that soy products, but not isoflavone supplements, significantly improved total cholesterol (TC), LDL-C, HDL-C, and TG [102]. It seems reasonable to conclude that current evidence from randomized control trials in humans do not sufficiently support the claim that isoflavone extracts can independently reduce CVD risk by modulating plasma lipids. It is possible that soy isoflavones may reduce CVD risk by protecting against the oxidation of LDL-C and the development of oxLDL, as opposed to a lipoprotein-lowering effect. However, preliminary studies indicate that conjugated isoflavone metabolites (the abundant form in circulation) are ineffective antioxidants in comparison to aglycone isoflavones [106].

The hypolipidemic effect of soy may be mediated through a synergistic interaction between its proteins and isoflavones. Animal studies have shown that serum lipids are augmented only when soy protein is consumed in combination with isoflavones [107,108]. A meta-analysis that examined the effect of soy protein consumed with varying doses of isoflavones found a lowering effect on TC, LDL-C, and TG, in addition to increased HDL-C among individuals with T2D [109]. There is also evidence suggesting that soy phytochemicals may elicit hypolipidemic effects. For example, hamsters that were fed a high-fat diet along with 200 mg/kg glyceollins (derivative molecules of isoflavones) experienced reduced plasma lipids and hepatic lipid content after 28 days of supplementation [110]. In rats, four-week supplementation with okara, a tofu by-product containing high amounts of protein and insoluble dietary fiber, resulted in lower serum TG and phospholipid concentrations [111]. Furthermore, it has been suggested that soy lipids such as lecithins and phospholipids could also potentiate the hypolipidemic effect of soy by inhibiting intestinal cholesterol absorption and promoting biliary cholesterol excretion [112]. High concentrations of phosphatidylcholine in soy lecithin may account for these modulatory effects on lipid metabolism by solubilizing cholesterol in the intestines, thereby restricting uptake by enterocytes [112]. An early study demonstrated that soybean phospholipid supplementation exerted direct effects on plasma lipids, producing a significant reduction in TC during supplementation, which was followed by a significant increase in cholesterol concentrations when the intervention was stopped [113]. Another study showed that a powder containing soy-derived stanols and lecithins lowered total- and LDL-C in a 10-week RCT of normo- and mildly hypercholesterolemic adults by reducing cholesterol absorption [114].

The combination of soy fibers and phospholipids with soy protein was found to have an additive hypocholesterolemic effect compared to when soy protein was consumed alone [115]. A low-glycemic index diet supplemented with soy protein and 4 g of soy phytosterols induced hypolipidemic effects in postmenopausal women, improved blood pressure, and lowered the Framingham risk score assessment for CHD in postmenopausal women [116]. Most recently, soy milk powder enriched with phytosterols was shown to significantly lower TC and LDL-C in mildly hypercholesterolemic Chinese adults after six months of daily intake; this effect was independent of the apolipoprotein E genotype [117], suggesting the observed effect was not attributed to inherent differences in cholesterol uptake. However, other factors such as the gut microbiota composition could mediate the cholesterol lowering effect of soy. For example, a recent study demonstrated increased gut microbial diversity in hamsters fed soy protein compared to dairy protein, which was related to an observed lipid lowering effect [118]. It is therefore apparent that the lipid lowering effect of soy is potentiated and mediated by inherent factors as well as constituents that act in synergy with protein.

### 3.4. Effects of Soy on Inflammation and Obesity

Inflammation is an innate immune response involving both intra- and extracellular pathways that utilize growth factors, cytokines, leukotrienes, and prostaglandins to attack foreign substances and eliminate them. In this regard, the development of atherosclerotic plaque is one such event that positively stimulates the immune response and results in a state of chronic inflammation [119]. Chronic inflammation can further lead to pathologies such as CVD by encouraging the transformation of healthy endothelial tissue into diseased tissue [119]. Available evidence supports an important role for soy isoflavones in mitigating markers of inflammation. For example, the isoflavone genistein protects against endothelial injury by down-regulating the expression of pro-inflammatory genes and inhibiting the production of reactive oxygen species (ROS) [120]. Studies in cell culture models have shown that genistein, but not daidzein, can mitigate cellular damage induced by peroxidase via an increase in glutathione peroxidase activity [121]. Daidzein has been shown to regulate the expression of inflammatory genes by modulating pathways involved in the activation of PPAR-α and -γ, and by inhibiting the c-Jun N-terminal kinase (JNK) pathway [122]. These findings are supported by preliminary human studies testing the independent effects of soy isoflavones. For example, a clinical trial involving prostate cancer patients showed that isoflavone-fortified bread lowered pro-inflammatory cytokines and chemokines [123]. In addition, increased isoflavone intake appeared to confer some protective effects when healthy young adults were challenged with an endotoxin to evoke an inflammatory response [124]. Further, isoflavone extracts taken by obese postmenopausal women improved serum concentrations of leptin, adiponectin, and tumor necrosis factor alpha (TNF-α) after supplementation for six months [125]. However, a six-month RCT involving 265 postmenopausal equol-producing Chinese women showed that significant improvements in plasma CRP concentrations were achieved only when whole soy, but not purified daidzein, was consumed [65].

Studies involving human volunteers have shown that soy protein also confers anti-inflammatory effects. For example, adults with T2D and nephropathy who consumed soy protein had significant reductions in serum CRP [126]. In addition, hemodialytic patients consuming 27 g/day of soy protein for six months had lower ratios of neutrophil:lymphocyte concentration, a marker of systemic inflammation [127], and a diet supplemented with soy nuts improved arterial stiffness in adults at risk for CVD [128]. The reasons for these anti-inflammatory effects are unclear; however, it has been postulated that the amino acid composition of soy protein may partly account for its ability to mitigate systemic inflammation [129]. Notably, glycine (an amino acid found abundantly in soy protein) was shown to promote antioxidant enzyme activity and inhibit inflammatory pathways in a rat model [130]. A particular challenge in interpreting the literature on soy and inflammation is that markers used to assess inflammation vary by study [65]. Moreover, several studies are conducted with postmenopausal women, which limit the generalizability of these results. Together, these limitations create difficulty in ascertaining the net effect of soy and its constituents using pooled analysis.

Obesity, now recognized as a state of disease, is closely linked with inflammation as excess adipose tissue (especially visceral adipocytes) secrete inflammatory cytokines and chemokines that can initiate and/or promote a pro-inflammatory state [131]. Obesity is also linked to an increase in circulating ROS, which can damage proteins and cellular organelles such as the mitochondria [132,133]. Additionally, low adiponectin concentrations, which are observed during obesity-associated inflammation, promote the development of insulin resistance, MetS, and CHD [131]. Given the epidemiological evidence linking soy consumption to healthier body weights [47], the specific mechanisms underlying this health benefit have been an active area of recent research.

Soy isoflavones are associated with anti-adipogenic effects, although evidence on this relies largely on data obtained from animal studies. For instance, long-term supplementation with isoflavones was shown to reduce body/visceral adipose tissue and serum leptin concentrations in adult female rats [134], and isoflavone extracts reduced body mass and plasma lipid concentrations in diet-induced obese male rats [135]. Another study using Huanjiang mini-pigs found that isoflavones regulated the expression of genes involved in lipid metabolism [136]. Similarly, mice consuming isoflavones extracts (0.15%) experienced reduced body weight, adipose mass, and TG concentration, possibly through the suppression of genes regulating PPAR-γ and SREBP-1c [67]. Isoflavone-induced upregulation of PPAR-α and PPAR-γ coactivator-1α (PGC-1α) was also postulated to promote the breakdown of fatty acids by inducing β-oxidation [137]. Controversially, some isoflavone studies have produced evidence that suggests isoflavone supplementation could have deleterious consequences: increases in adipose tissue were observed among male mice fed a low-fat diet supplemented with purified genistein [138], and TC and leptin concentrations significantly increased when mice were supplemented with 0.45% isoflavone extracts [139]. Further studies have demonstrated other benefits such as reduced body and liver mass, blood glucose, TC, and serum leptin concentrations in mice fed a high-fat diet supplemented with black soybean [140].

Studies examining the effect of soy on weight loss in obese humans are limited. In one of these studies, obese postmenopausal women ingesting 75 mg isoflavone conjugates daily experienced reductions in trunk fat mass after 12 months of supplementation [141]. In contrast, a multi-center dose-response study showed that obese postmenopausal women who ingested soy isoflavone tablets containing 80 or 120 mg isoflavone conjugates daily did not experience significant improvements in body composition [142]. However, in adults (18–95 years), obesity was associated with the status of *O*DMA non-producer, which suggests additional work is required to clarify the role of isoflavone-metabolizing-phenotype on the bioactivity of soy isoflavones consumed during intervention studies [143].

The effect of other soy components on obesity has also been examined. One study found that biscuits fortified with soy fiber significantly lowered body weight, BMI and total- and LDL-C in healthy adults [28], although a similar effect was not observed when obese participants with non-alcoholic fatty liver disease consumed a low-carbohydrate diet supplemented with soy nuts [144]. Participants enrolled in a weight loss exercise and nutrition program experienced greater, but not statistically significant, weight loss when assigned to a whole soy group compared to the wheat supplemented group despite engaging in equal amounts of exercise [145].

The efficacy of soy protein in improving body composition has been examined in a few human trials and the overall results suggest no intervention effects; moreover, it does not appear to be an effective weight loss aid. For example, postmenopausal obese women consuming a calorie-restricted diet supplemented with soy protein did not lose significantly more weight compared to a diet without soy [146]. Another RCT revealed that soy protein was not as effective as milk protein in lowering visceral and subcutaneous fat mass [147], and postmenopausal women did not experience significant reductions in BMI or waist-to-hip ratio after consuming isoflavone-rich soy protein daily for 12 months [148]. However, the addition of black soy protein led to more significant declines in body weight, body fat mass and leptin in 64 overweight/obese participants [149], and in a 12-month exercise and nutrition intervention study, obese women lost significantly more body weight with a soy yogurt meal replacement compared to control [150].

## 4. Contribution of Soy Based Foods to Satiety

The effect of consumption of soy and soy products on satiety-related measures has been explored in a few studies. In one such study, soy isoflavone supplementation did not significantly influence energy intake, body weight, nor serum ghrelin concentrations in healthy postmenopausal women after eight weeks [151]. Similarly, isoflavone tablets did not significantly affect plasma concentrations of insulin, leptin, ghrelin, and adiponectin in postmenopausal women after 12 months [142]. In addition, soy flour (27.3%) incorporated into a pretzel-like bread did not significantly alter satiety scores [152], and there were no significant differences in appetite, satiety, or food consumption when identical portions of beef or soy protein were consumed [153]. Similarly, obese men consuming a soy-based, high-protein diet did not experience more weight loss, nor was food intake reduced compared to men consuming a diet with beef [154]. Overweight and obese men who consumed an isoflavone-free soy protein preload 30 min before lunch had reduced appetite and caloric intake after 12 weeks, but whey protein exhibited more substantial effects [155]. However, muffins made with soy flour elicited greater feeling of fullness scores among mildly hypercholesterolemic overweight adults, compared to a wheat muffin supplemented with whey protein [156]. In another study, snacks made with soy protein isolates led to greater reductions in appetite and reduced intake of sugary snacks in the evening among adolescents [157], and a breakfast high in soy protein reduced serum appetite hormones including ghrelin and protein YY [158]. Obese mice also exhibited greater satiety while consuming whey protein as compared to soy protein [159]. Altogether, the limited evidence on the effect of soy and satiety appears to suggest that soy protein, isoflavones, and other soy constituents do not elicit a satiating effect any greater than what is observed with a comparable quantity of animal protein. A summary findings from randomized control trials based on the impact of soy on obesity and satiety is presented in Table 3.

## 5. Conclusions

Isoflavones and their metabolites appear to improve blood pressure, glycemic control, obesity, and inflammation. Evidence on the hypotensive effects of other soy components such as protein, fiber, lecithins, and saponins is limited by a dearth of available RCTs and current observations suggest that these components do not elicit significant hypotensive effects. However, these constituents may act in synergy with soy protein to modulate plasma lipids to a greater extent than when soy protein is independently consumed. Few human studies have examined the effect of minor soy constituents on glycemic control, despite convincing mechanistic evidence supporting these effects. The anti-adipogenic effects of isoflavones have been tested mostly in animal models, with promising results; however, soy protein does not appear to improve body composition greater than what is achieved with comparable levels of milk protein. A limited body of literature suggests that soy protein and other constituents may enhance satiety. Together, these findings demonstrate the importance of several soybean bioactive compounds in reducing CVD risk that complement the well-established effects of soy protein. Additional RCTs examining the independent effects of these components, and the role of the microbiome in mitigating these effects, are needed to support the direct health benefits of these novel bioactive compounds.

## Figures and Tables

**Table 1:** Arginine and lysine content ^1^ of select foods.

Food	Arginine	Lysine	PDCAAS ^2^
Soy protein isolate	6670	5327	1.00
Peanuts, dry-roasted	2832	850	0.52
Almonds, dry-roasted	2444	563	0.23
Black beans, boiled	549	608	0.75
Milk, 2% (cow)	94	276	1.00
Egg, hard-boiled	755	904	1.00
Beef, chuck, braised	2054	2748	0.92

^1^ Adopted from the US Department of Agriculture National Nutrient Database [75], and expressed as mg/100 g edible portion; ^2^ The Protein Digestibility Corrected Amino Acid Score (PDCAAS) is the WHO preferred method for evaluating protein quality (scored numerically, 0–1) on the basis of amino acid composition relative to human requirements [76]. Scores adopted from previously published work [77,78,79].

**Table 2:** Summary findings from randomized control trials based on the impact of soy on hyperglycemia.

Study	Sample Size & Population	Duration	Treatment	Dose; Format	Results	Conclusions
[89]	389 osteopenic postmenopausal women	2 years, parallel RCT	Genistein extract with calcium and Vitamin D3 supplement compared to placebo	Purified supplement (54 mg/day)	Significant reductions in FBG, insulin, HOMA-IR, fibrinogen, F2-isoprostanes, soluble intercellular adhesion molecule-1, and soluble vascular cellular adhesion molecule-1	Genistein supplements improve markers of glycemic control and other markers of CVD risk
[97]	42 postmenopausal women with MetS	8 weeks, randomized crossover trial	Control diet (Dietary Approaches to Stop Hypertension, DASH); DASH diet with red meat replaced with soy nuts; DASH diet with red meat replaced with soy protein	30 g soy nut = 1 serving read meat; 30 g soy protein = 1 serving red meat. Normal diet for 3 weeks followed by all 3 diets for 8 weeks each, with 4-week washout period in between each diet	Soy nuts reduced HOMA-IR and fasting plasma glucose more than soy protein (*p* < 0.01 for both factors) and control (*p* < 0.01 for both factors)	Soy nuts have greater role in attenuating blood glucose response markers compared to soy protein in postmenopausal women with MetS
[96]	75 women with MetS, age 60–70	12 weeks, parallel RCT	Soy nuts; textured soy protein (TSP)	35 g/day soy nut; 35 g/day textured soy protein	Compared to control, serum FBG, HOMA-IR and insulin were lower in soy-nut group (*p* < 0.05, *p* < 0.1, *p* < 0.05) and the mean changes were higher in soy nut group vs. TSP (*p* < 0.001)	Soy nut leads to greater reductions in markers of glycemic control than soy protein
[88]	203 postmenopausal women, age 48–62.	1 year parallel RCT	Calcium tablet with isoflavones	40 or 80 mg isoflavones/day compared to calcium with 0 mg isoflavones	Both treatment groups significantly lowered FBG. No dose-response effect. No effect on lipids	Isoflavone supplementation favourably influences FBG in postmenopausal women
[90]	180 postmenopausal women with hyperglycemia	6 months parallel RCT	Soy protein isolate with or without isoflavone conjugates	15 g soy protein and 100 mg isoflavones, 15 g milk protein and 100 mg isoflavones, or 15 g milk protein	No significant improvements in fasting and 2-h postload glucose, fasting and postload insulin, glycated serum protein, HOMA-IR. and beta-cell function	Soy protein isolate with or without isoflavones does not improve glycemic control and insulin sensitivity
[99]	20 healthy males and females	1 day; within-subject design with 1 week intervals in between interventions	Intervention (1) Bar-type cake made of whole soy powder; (2) cooked paddy-rice; (3) cooked paddy rice with whole soy powder cake	114 g whole soy powder cake containing 50 g carbohydrates, 144 g cooked paddy-rice containing 50 g carbohydrates; 144 g cooked paddy-rice with 60 g whole soy powder sake	(1) Blood glucose and insulin levels were lower than control (2) Blood was lower, while insulin levels were increased slightly	Postprandial blood glucose and insulin response may be improved with whole soy powder food; beneficial effects of combining soy products with carbohydrate-rich foods are less clear
[87]	120 postmenopausal women with Metabolic Syndrome (60 received treatment; 60 received placebo)	1 year, randomized, double-blind, placebo-controlled trial	Genistein	54 mg/day in 2 tablets	Fasting blood glucose, fasting insulin and HOMA-IR were significantly decreased in the treatment group (*p* < 0.001)	Soy isoflavone, genistein, significantly lowers hyperglycemia and reduces insulin resistance
[98]	10 type 2 diabetes patients in the 80 kcal meal tolerance test (Study 1); 11 diabetic patients in the 592 kcal meal tolerance test (Study 2)	1 day, crossover study	Soybean nutrition bar	Study 1: 1 soybean nutrition bar containing 7.0 g carbohydrates, 4.3 g fat, 1.9 g fiber, 2.7 g protein, 8.2 mg isoflavones Study 2: 4.3 soybean nutrition bars, each containing 50.9 g carbohydrates, 31.3 g fat, 14.4 g fiber, 19.6 g protein, 14 mg isoflavones	Blood glucose was lower in both studies with the soybean nutrition bar intervention (*p* < 0.001); Insulin AUC was lower than the control in study 2 only; no significant changes in blood TGs and non-esterified fatty acids between treatment and control groups in either study	Soybean nutrition bars may have a role in preventing postprandial hyperglycemia in patients with type 2 diabetes compared to isocaloric cookies
[91]	165 women with impaired glucose regulation, aged 30–70	24 week parallel RCT	Soy protein with or without daidzein or genistein extract	10 g soy protein without isoflavones, or with 50 mg daidzein, or 50 mg genistein	No significant changes in FBG, 2-h glucose, HbA1c, fasting, and 2-h insulin, AUC of glucose and insulin.	Purified extracts of daidzein and genistein do not improve glycemic control and insulin sensitivity

AUC = area under blood glucose response curve; CVD = cardiovascular disease; FBG = fasting blood glucose; HBA1c = glycated hemoglobin; HOMA-IR = homeostatic model assessment of insulin resistance; RCT = randomized controlled trial; MetS = metabolic syndrome.

**Table 3:** Summary findings from randomized control trials based on the impact of soy on obesity and satiety.

Study	Sample Size & Population	Duration	Treatment	Dose; Format	Results	Conclusions
[146]	25 abdominally obese men and women	12 weeks, parallel RCT	Soy protein meal replacement	4 replacement meals/day: 44 g soy protein, 60–135 mg isoflavones/day	No significant variations in body composition (including weight, BMI, % fat and % lean mass) or cardiometabolic risk factors were observed in the soy replacement meal compared to control	Soy protein affects weight loss to same extent as other proteins
[28]	39 overweight and obese adults	Daily breakfast for 12 weeks	Soy fiber supplemented biscuits	100 g soy fiber/day	LDL-C, TC and BMI decreased after 12 weeks (*p* < 0.05)	Soy fiber supplementation may help in weight management and lowering cholesterol levels
[144]	45 patients with non-alcoholic fatty liver disease	8 weeks, parallel RCT	3 diets: low-calorie; low- calorie, low carbohydrate; low-calorie, low soy diet	30 g soy nuts containing: 7 g fat, 9 g fiber, 11.3 g protein, 10 mg sodium, 102 mg phytoestrogens	Soy group had greatest decline in serum liver enzymes and fibrinogens, and malondialdehyde. There were no changes in BMI or weight between the two groups	Soy may mitigate inflammation when combined with a low-calorie diet, but may not affect weight loss in those with non-alcoholic fatty liver disease.
[158]	11 overweight and obese men	1 day, parallel RCT	Soy protein breakfast meal replacement, followed by standardized lunch 4 h later	Meal replacement, containing 28.7 soy protein (34.6 g total protein), 19.8 g carbohydrate	Meal replacement group had lower glucose levels, glucose and insulin AUC, ghrelin concentrations following breakfast; Fat oxidation was lower after lunch	Soy protein could have a potential hypoglycemic effect, as well as mediating metabolic risk factors and improving weight management
[150]	380 women, BMI of 30–40 kg/m^2^, 76 of whom took the soy meal replacement	12 months, with meal replacement taken for a maximum of 3 months (most did so during first 3 months of study)	Soy-based meal replacement (in addition to baseline lifestyle program administered to both control and treated groups)	Soy-yogurt-honey product: 83% soy-protein isolate, 17% milk protein	Weight loss was greater in meal replacement group (*p* = 0.1); health-related quality of life scores also increased more compared to control	Soy protein consumption may enhance weight loss when combined with a lifestyle intervention program focusing on diet and physical activity
[149]	64 overweight/obese subjects	12 weeks, parallel RCT	Black soy peptide (BSP) supplementation	4.5 g/day in tablet form	After 12 weeks, BSP group had statistically significant decrease in body weight, body fat mass, body fat % and plasma leptin (vs. no change in placebo); no change in inflammatory markers or lipid profiles between groups	Black soy peptide seems to have a role in weight and fat mass regulation in overweight subjects, perhaps by altering leptin levels
[156]	116 healthy men and women	6 weeks, parallel RCT	Soy muffins (vs. control wheat muffins)	12.5 g soy protein muffin; 2 muffins/day	Higher fullness scores in soy muffin group on a Visual Analog Scale (*p* = 0.002) vs. control group	Replacing wheat flour with soy flour may increase perceived satiety
[145]	38 obese men and women	8 weeks, parallel RCT	Whole soy powder bar (vs. wheat control)	1 bar eaten 1–2 h before dinner daily	Compared to control, soy group had lower BMI, waist circumference and body fat % (*p* < 0.05). No significant differences in weight loss, TC, LDL = C, or insulin were found between the two groups	Whole soy can complement the benefits of a weight-loss program; whether it does so by mediating glycemic response remains unclear based on this study.
[147]	48 obese Japanese adults	20 weeks, parallel RCT	Soy protein	Soy protein intervention containing 12 g soy protein, 9 g milk protein; consumed at breakfast	Visceral and subcutaneous fat, body weight and BMI decreased significantly after 20 weeks of milk protein ingestion, but not after ingestion of soy protein containing milk protein	Milk protein has a more substantial impact on markers of weight loss compared to a combination of milk and soy protein

AUC = area under blood glucose response curve; BMI = body mass index; LDL-C = low density lipoprotein cholesterol; RCT = randomized control trial; TC = total cholesterol.

## Abbreviations

AUC	area under the curve
BMI	body mass index
cAMP	cyclic adenosine monosphosphate
CHD	coronary heart disease
CRP	C-reactive protein
CVD	cardiovascular disease
FBG	fasting blood glucose
HDL-C	high density lipoprotein cholesterol
HOMA-IR	homeostatic model assessment of insulin resistance
LDL-C	low density lipoprotein cholesterol
LDLR	low density lipoprotein cholesterol receptor
oxLDL	oxidized LDL cholesterol
MetS	metabolic syndrome
NO	nitric oxide
*O*DMA	*O*-desmethylangolensin
PPAR	peroxisome proliferator activated receptor
PKA	protein kinase A
RCT	randomized controlled trial
ROS	reactive oxygen species
TC	total cholesterol
TNF-α	tumor necrosis factor alpha
TG	triglycerides

## References

[1] World Health Organization (2011). Global Atlas on Cardiovascular Disease Prevention and Control: Policies, Strategies and Intervention.

[2] Ross R. (1999). Atherosclerosis—An inflammatory disease. N. Engl. J. Med..

[3] Kannel W.B. (1996). Blood pressure as a cardiovascular risk factor: Prevention and treatment. JAMA.

[4] Duckworth W.C. (2001). Hyperglycemia and cardiovascular disease. Curr. Atheroscler. Rep..

[5] Schulze P.C., Lee R.T. (2005). Oxidative stress and atherosclerosis. Curr. Atherscler. Rep..

[6] Bouchenak M., Lamri-Senhadji M. (2013). Nutritional quality of legumes, and their role in cardiometabolic risk prevention: A review. J. Med. Food.

[7] Mattson J.W., Sun C., Koo W.W. Analysis of the World Oil Crops Market. http://ageconsearch.umn.edu/handle/23621.

[8] Singh P., Kumar R., Sabapathy S.N., Bawa A.S. (2008). Functional and edible uses of soy protein products. CRFSFS.

[9] Torres N., Torre-Villalvazo I., Tovar A.R. (2006). Regulation of lipid metabolism by soy protein and its implication in diseases mediated by lipid disorders. J. Nutr. Biochem..

[10] Maki K.C., Butteiger D.N., Rains T.M. (2010). Effects of soy protein on lipoprotein lipids and fecal bile acid excretion in men and women with moderate hypercholesterolemia. J. Clin. Lipidol..

[11] Anderson J.W., Johnston B.M., Cook-Newell M.E. (1995). Meta-analysis of the effects of soy-protein intake on serum lipids. N. Engl. J. Med..

[12] Weggemans R.M., Trautwein E.A. (2003). Relation between soy-associated isoflavones and LDL and HDL cholesterol concentrations in humans: A meta-analysis. Eur. J. Clin. Nutr..

[13] Yueng J., Yu T.F. (2003). Effects of isoflavones (soy phyto-estrogens) on serum lipids: A meta-analysis of randomized controlled trials. Nutr. J..

[14] Zhuo X.-G., Melby M.K., Watanabe S. (2004). Soy isoflavone intake lowers serum LDL cholesterol: A meta-analysis of 8 randomized controlled trials in humans. J. Nutr..

[15] Zhan S., Ho S.C. (2005). Meta-analysis of the effects of soy-protein containing isoflavones on the lipid profile. Am. J. Clin. Nutr..

[16] Reynolds K., Chin A., Lees K.A., Nguyen A., Bujnowski D., He J. (2006). A meta-analysis of the effect of soy protein supplementation on serum lipids. Am. J. Cardiol..

[17] Taku K., Umegaki K., Sato Y., Taki Y., Endoh K., Watanabe S. (2007). Soy isoflavones lower serum total and LDL cholesterol in humans: A meta-analysis of 11 randomized controlled trials. Am. J. Clin. Nutr..

[18] Taku K., Umegaki K., Ishimi Y., Watanabe S. (2008). Effects of extracted soy isoflavones alone on blood total and LDL cholesterol: Meta-analysis of randomized controlled trials. Ther. Clin. Risk Manag..

[19] Harland J.I., Haffner T.A. (2008). Systematic review, meta-analysis and regression of randomised controlled trials reporting an association between an intake of circa 25 g soya protein per day and blood cholesterol. Atherosclerosis.

[20] Jenkins D.J., Mirrahimi A., Srichaikul K., Berryman C.E., Wang L., Carleton A., Abdulnour S., Sievenpiper J.L., Kendall C.W., Kris-Etherton P.M. (2010). Soy protein reduces serum cholesterol by both intrinsic and food displacement mechanisms. J. Nutr..

[21] Benkhedda K., Boudrault C., Sinclair S.E., Marles R.J., Xiao C.W., Underhill L. (2014). Health Canada’s proposal to accept a health claim about soy products and cholesterol lowering. Int. Food Risk Anal. J..

[22] U.S. Food & Drug Administration. http://www.fda.gov/Food/GuidanceRegulation/GuidanceDocumentsRegulatoryInformation/LabelingNutrition/ucm064919.htm.

[23] Health Canada: Summary of Health Canada’s Assessment of a Health Claim about Soy Protein and Cholesterol Lowering. http://www.hc-sc.gc.ca/fn-an/label-etiquet/claims-reclam/assess-evalu/soy-protein-cholesterol-eng.php.

[24] European Food Safety Authority Scientific Opinion on the Substantiation of a Health Claim Related to Isolated Soy Protein and Reduction of Blood LDL-Cholesterol Concentrations Pursuant to Article 14 of Regulation (EC) No 1924/2006. http://www.efsa.europa.eu/en/efsajournal/pub/2555.

[25] Dixit A.K., Antony J.I.X., Sharma N.K., Tiwari R.K. Soybean Constituents and Their Functional Benefits. http://www.trnres.com/ebook/uploads/tiwari/T_1302158885Tiwari-12.pdf.

[26] McCue P., Kalidas S. (2004). Health benefits of soy isoflavonoids and strategies for enhancement: A review. Crit. Rev. Food Sci. Nutr..

[27] Manach C., Scalbert A., Morand C., Rémésy C., Jiménez L. (2004). Polyphenols: Food sources and bioavailability. Am. J. Clin. Nutr..

[28] Hu X., Gao J., Zhang Q., Fu Y., Li K., Zhu S., Li D. (2013). Soy fiber improves weight loss and lipid profile in overweight and obese adults: A randomized controlled trial. Mol. Nutr. Food Res..

[29] McGraw N.J., Krul E.S., Grunz-Borgmann E., Parrish A.R. (2016). Soy-based renoprotection. World J. Nephrol..

[30] Omoni A.O., Aluko R.E. (2005). Soybean foods and their benefits: Potential mechanisms of action. Nutr. Rev..

[31] Rebholz C.M., Friedman E.E., Powers L.J., Arroyave W.D., He J., Kelly T.N. (2012). Systematic reviews and meta- and pooled analyses dietary protein intake and blood pressure: A meta-analysis of randomized controlled trials. Am. J. Epidemiol..

[32] Barnes S., Prasain J., D’Alessandro T., Arabshahi A., Botting N., Lila M.A., Jackson G., Janle E.M., Weaver C.M. (2011). The metabolism and analysis of isoflavones and other dietary polyphenols in foods and biological systems. Food Funct..

[33] Baber R.J., Preedy V.R. (2011). Phytoestrogens in health: The role of isoflavones. Isoflavones: Chemistry, Analysis, Function and Effects.

[34] Shimazu T., Kuriyama S., Hozawa A., Ohmori K., Sato Y., Nakaya N., Nishino Y., Tsubono Y., Tsuji I. (2007). Dietary patterns and cardiovascular disease mortality in Japan: A prospective cohort study. Int. J. Epidemiol..

[35] Nagata C., Wada K., Tamura T., Konishi K., Goto Y., Koda S., Kawachi T., Tsuji M., Nakamura K. (2016). Dietary soy and natto intake and cardiovascular disease mortality in Japanese adults: The Takayama study. Am. J. Clin. Nutr..

[36] Reinwald S., Akabas S.R., Weaver C.M. (2010). Whole versus the piecemeal approach to evaluating soy. J. Nutr..

[37] Setchell K.D.R., Clerici C. (2010). Equol: History, chemistry, and formation. J. Nutr..

[38] Liu Z.M., Ho S.C., Chen Y.M., Liu J., Woo J. (2014). Cardiovascular risks in relation to daidzein metabolizing phenotypes among Chinese postmenopausal women. PLoS ONE.

[39] Vedrine N., Mathey J., Morand C., Brandolini M., Davicco M.-J., Guy L., Remesy C., Coxam V., Manach C. (2006). One-month exposure to soy isoflavones did not induce the ability to produce equol in postmenopausal women. Eur. J. Clin. Nutr..

[40] Vergne S., Sauvant P., Lamothe V., Chantre P. (2009). Influence of ethnic origin (Asian v. Caucasian) and background diet on the bioavailability of dietary isoflavones. Br. J. Nutr..

[41] Yamori Y. (2004). Worldwide epidemic of obesity: Hope for Japanese diets. Clin. Exp. Pharmacol. Physiol..

[42] Wu S.H., Shu X.O., Chow W.H., Xiang Y.B., Zhang X., Li H.L., Cai Q., Ji B., Cai H., Rothman N. (2013). Soy food intake and circulating levels of inflammatory markers in Chinese women. J. Acad. Nutr. Diet..

[43] Borgi L., Muraki I., Satija A., Willett W.C., Rimm E.B., Forman J.P. (2016). Fruit and vegetable consumption and the incidence of hypertension in three prospective cohort studies. Hypertension.

[44] Golzarand M., Bahadoran Z., Mirmiran P., Sadeghian-Sharif S., Azizi F. (2015). Dietary phytochemical index is inversely associated with the occurrence of hypertension in adults: A 3-year follow-up (the Tehran Lipid and Glucose Study). Eur. J. Clin. Nutr..

[45] Tatsumi Y., Morimoto A., Deura K., Mizuno S., Ohno Y., Watanabe S. (2013). Effects of soybean product intake on fasting and postload hyperglycemia and type 2 diabetes in Japanese men with high body mass index: The Saku Study. J. Diabetes Investig..

[46] Nanri A., Mizoue T., Takahashi Y., Kirii K., Inoue M., Noda M., Tsugane S. (2010). Soy product and isoflavone intakes are associated with a lower risk of type 2 diabetes in overweight Japanese women. J. Nutr..

[47] Mueller N.T., Odegaard A.O., Gross M.D., Koh W.P., Mimi C.Y., Yuan J.M., Pereira M.A. (2012). Soy intake and risk of type 2 diabetes mellitus in Chinese Singaporeans. Eur. J. Nutr..

[48] Ding M., Pan A., Manson J.E., Willett W.C., Malik V., Rosner B., Giovannucci E., Hu F.B., Sun Q. (2016). Consumption of soy foods and isoflavones and risk of type 2 diabetes: A pooled analysis of three US cohorts. Eur. J. Clin. Nutr..

[49] Morimoto Y., Steinbrehcer A., Kolonel L., Maskarinec G. (2011). Soy consumption is not protective against diabetes in Hawaii: The multiethnic cohort. Eur. J. Clin. Nutr..

[50] Lou D., Li Y., Yan G., Bu J., Wang H. (2016). Soy consumption with risk of coronary heart disease and stroke: A meta-analysis of observational studies. Neuroepidemiology.

[51] Pujol T.J., Tucker J.E., Nelms M., Sucher K., Long S. (2007). Diseases of the cardiovascular system. Nutrition Therapy and Pathophysiology.

[52] Tanira M.O., Al Balushi K.A. (2005). Genetic variations related to hypertension: A review. J. Hum. Hypertens..

[53] Jackson R.L., Greiwe J.S., Schwen R.J. (2011). Emerging evidence of the health benefits of S-equol, an estrogen receptor β-agonist. Nutr. Rev..

[54] Colacurci N., Chiantera A., Fornaro F., de Novellis V., Manzella D., Arciello A., Chiantera V., Improta L., Paolisso G. (2005). Effects of soy isoflavones on endothelial function in healthy postmenopausal women. Menopause.

[55] Patten G.S., Abeywardena M.Y., Bennett L.E. (2016). Inhibition of angiotensin converting enzyme, angiotensin II receptor blocking, and blood pressure lowering bioactivity across plant families. Crit. Rev. Food Sci. Nutr..

[56] Taku K., Lin N., Cai D., Hu J., Zhao X., Zhang Y., Wang P., Melby M.K., Hooper L., Kurzer M.S. (2010). Effects of soy isoflavone extract supplements on blood pressure in adult humans: Systematic review and meta-analysis of randomized placebo-controlled trials. J. Hypertens..

[57] Liu X.X., Li S.H., Chen J.Z., Sun K., Wang X.J., Wang X.G., Hui R.T. (2012). Effect of soy isoflavones on blood pressure: A meta-analysis of randomized controlled trials. Nutr. Metab. Cardiovasc. Dis..

[58] Garcia Garcia M.A., Arenas R., Martinez C.A., Perez L.L. (2016). Usefulness of phytoestrogens in treatment of arterial hypertension: Systematic review and meta-analysis. Arch. Clin. Hypertens..

[59] Hügel H.M., Jackson N., May B., Zhang A.L., Xue C.C. (2016). Polyphenol protection and treatment of hypertension. Phytomedicine.

[60] Hazim S., Curtis P.J., Schär M.Y., Ostertag L.M., Kay C.D., Minihane A.-M., Cassidy A. (2016). Acute benefits of the microbial-derived isoflavone metabolite equol on arterial stiffness in men prospectively recruited according to equol producer phenotype: A double-blind randomized controlled trial. Am. J. Clin. Nutr..

[61] Acharjee S., Zhou J., Elajami T.K., Welty F.K. (2015). Effect of soy nuts and equol status on blood pressure, lipids and inflammation in postmenopausal women stratified by metabolic syndrome status. Metabolism.

[62] Liu Z., Ho S.C., Chen Y., Tomlinson B., Ho S., To K., Woo J. (2015). Effect of whole soy and purified daidzein on ambulatory blood pressure and endothelial function—A 6-month double-blind, randomized controlled trial among Chinese postmenopausal women with prehypertension. Eur. J. Clin. Nutr..

[63] Nishibori N., Kishibuchi R., Morita K. (2017). Soy pulp extract inhibits angiotensin I-converting enzyme (ACE) activity in vitro: Evidence for its potential hypertension-improving action. J. Diet. Suppl..

[64] Van der Kuil W.A., Engberink M.F., Brink E.J., van Baak M.A., Bakker S.J.L., Navis G., van’t Veer P., Geleijnse J.M. (2010). Dietary protein and blood pressure: A systematic review. PLoS ONE.

[65] Liu Z.M., Ho S.C., Chen Y.M., Woo J. (2013). Effect of soy protein and isoflavones on blood pressure and endothelial cytokines: A 6-month randomized controlled trial among postmenopausal women. J. Hypertens..

[66] Welty F.K., Lee K.S., Lew N.S., Zhou J.R. (2007). Effect of soy nuts on blood pressure and lipid levels in hypertensive, prehypertensive, and normotensive postmenopausal women. Arch. Intern. Med..

[67] Zang Y., Zhang L., Igarashi K., Yu C. (2015). The anti-obesity and anti-diabetic effects of kaempferol glycosides from unripe soybean leaves in high-fat-diet mice. Food Funct..

[68] Mohammadifard N., Salehi-Abargouei A., Salas-Salvadó J., Guasch-Ferré M., Humphries K., Sarrafzadegan N. (2015). The effect of tree nut, peanut, and soy nut consumption on blood pressure: A systematic review and meta-analysis of randomized controlled clinical trials. Am. J. Clin. Nutr..

[69] Moghaddam A.S., Entezari M.H., Iraj B., Gholamreza A., Zahabi E.S., Maracy M.R. (2014). The effects of soy bean flour enriched bread intake on anthropometric indices and blood pressure in type 2 diabetic women: A crossover randomized controlled clinical trial. Int. J. Endocrinol..

[70] Evans M., Njike V.Y., Hoxley M., Pearson M., Katz D.L. (2007). Effect of soy isoflavone protein and soy lecithin on endothelial function in healthy postmenopausal women. Menopause.

[71] Moncada S., Higgs A. (1993). The L-arginine-nitric oxide pathway. N. Engl. J. Med..

[72] Dong J.-Y., Qin L.-Q., Zhang Z., Zhao Y., Wang J., Arigoni F., Zhang W. (2011). Effect of oral L-arginine supplementation on blood pressure: A meta-analysis of randomized, double-blind, placebo-controlled trials. Am. Heart J..

[73] Bai Y., Sun L., Yang T., Sun K., Chen J., Hui R. (2009). Increase in fasting vascular endothelial function after short-term oral L-arginine is effective when baseline flow-mediated dilation is low: Meta-analysis of randomized controlled trials. Am. J. Clin. Nutr..

[74] Vasdev S., Gill V. (2008). The antihypertensive effect of arginine. Int. J. Angiol..

[75] United States Department of Agriculture Agricultural Research Service: USDA Food Composition Databases. https://ndb.nal.usda.gov/ndb/search/list.

[76] Sarwar G., McDonough F.E. (1990). Evaluation of protein digestibility-corrected amino acid score method for assessing protein quality of foods. J. Assoc. Off. Anal. Chem..

[77] Hughes G.J., Ryan D.J., Mukherjea R., Schasteen C.S. (2011). Protein digestibility-corrected amino acid scores (PDCAAS) for soy protein isolates and concentrate: Criteria for evaluation. J. Agric. Food Chem..

[78] Ahrens S., Venkatachalam M., Mistry A.M., Lapsley K., Sathe S.K. (2005). Almond (*Prunus dulcis* L.) protein quality. Plant Foods Hum. Nutr..

[79] Hoffman J.R., Falvo M.J. (2004). Protein- which is best?. J. Sports Sci. Med..

[80] World Health Organization (2006). Definition and Diagnosis of Diabetes Mellitus and Intermediate Hyperglycaemia: Report of a WHO/IDF Consultation.

[81] Younis N., Charlton-Menys V., Sharma R., Soran H., Durrington P.N. (2009). Glycation of LDL in non-diabetic people: Small dense LDL is preferentially glycated both in vivo and in vitro. Atherosclerosis.

[82] Yang W., Wang S., Li L., Liang Z., Wang L. (2011). Genistein reduces hyperglycemia and islet cell loss in a high-dosage manner in rats with alloxan-induced pancreatic damage. Pancreas.

[83] Fu Z., Gilbert E.R., Pfeiffer L., Zhang Y., Fu Y., Liu D. (2012). Genistein ameliorates hyperglycemia in a mouse model of nongenetic type 2 diabetes. Appl. Physiol. Nutr. Metab..

[84] Harini R., Ezhumalai M., Pugalendi K.V. (2012). Antihyperglycemic effect of biochanin A, a soy isoflavone, on streptozotocin-diabetic rats. Eur. J. Pharmacol..

[85] Gilbert E.R., Liu D. (2013). Anti-diabetic functions of soy isoflavone genistein: Mechanisms underlying effects on pancreatic β-cell function. Food Funct..

[86] Ademiluyi A.O., Oboh G. (2013). Soybean phenolic-rich extracts inhibit key-enzymes linked to type 2 diabetes (α-amylase and α-glucosidase) and hypertension (angiotensin I converting enzyme) in vitro. Exp. Toxicol. Pathol..

[87] Squadrito F., Marini H., Bitto A., Altavilla D., Polito F., Adamo E.B., D’Anna R., Arcoraci V., Burnett B.P., Minutoli L. (2013). Genistein in the metabolic syndrome: Results of a randomized clinical trial. J. Clin. Endocrinol. Metab..

[88] Ho S.C., Chen Y.M., Ho S.S., Woo J.L. (2007). Soy isoflavone supplementation and fasting serum glucose and lipid profile among postmenopausal Chinese women: A double-blind, randomized, placebo-controlled trial. Menopause.

[89] Atteritano M., Marini H., Minutoli L., Polito F., Bitto A., Altavilla D., Mazzaferro S., D’Anna R., Cannata M.L., Gaudio A. (2007). Effects of the phytoestrogen genistein on some predictors of cardiovascular risk in osteopenic, postmenopausal women: A two-year randomized, double-blind, placebo-controlled study. J. Clin. Endocrinol. Metab..

[90] Liu Z.M., Chen Y.M., Ho S.C., Ho Y.P., Woo J. (2010). Effects of soy protein and isoflavones on glycemic control and insulin sensitivity: A 6-month, randomized, placebo-controlled trial in postmenopausal Chinese women with prediabetes or untreated early diabetes. Am. J. Clin. Nutr..

[91] Ye Y.B., Chen A.L., Lu W., Zhuo S.Y., Liu J., Guan J.H., Deng W.P., Fang S., Li Y.B., Chen Y.M. (2015). Daidzein and genistein fail to improve glycemic control and insulin sensitivity in Chinese women with impaired glucose regulation: A double-blind, randomized, placebo-controlled trial. Mol. Nutr. Food Res..

[92] Ricci E., Cipriani S., Chiaffarino F., Malvezzi M., Parazzini F. (2010). Effects of soy isoflavones and genistein on glucose metabolism in perimenopausal and postmenopausal non-Asian women: A meta-analysis of randomized controlled trials. Menopause.

[93] Fang K., Dong H., Wang D., Gong J., Huang W., Lu F. (2016). Soy isoflavones and glucose metabolism in menopausal women: A systematic review and meta-analysis of randomized controlled trials. Mol. Nutr. Food Res..

[94] Zhang X., Zhang Y., Chi M. (2016). Soy protein supplementation reduces clinical indices in type 2 diabetes and metabolic syndrome. Yonsei Med. J..

[95] Liu Z.M., Chen Y.M., Ho S.C. (2011). Effects of soy intake on glycemic control: A meta-analysis of randomized controlled trials. Am. J. Clin. Nutr..

[96] Bakhtiary A., Yassin Z., Hanachi P., Rahmat A., Ahmad Z., Halalkhor S., Firouzjahi A.R. (2011). Evaluation of the Oxidative Stress and Glycemic Control Status in Response to Soy in Older Women with the Metabolic Syndrome. Iran. Red Crescent Med. J..

[97] Azadbakht L., Kimiagar M., Mehrabi Y., Esmaillzadeh A., Padyab M., Hu F.B., Willett W.C. (2007). Soy inclusion in the diet improves features of the metabolic syndrome: A randomized crossover study in postmenopausal. Am. J. Clin. Nutr..

[98] Urita Y., Noda T., Watanabe D., Iwashita S.O.H., Hamada K., Sugimoto M. (2012). Effects of a soybean nutrition bar on the postprandial blood glucose and lipid levels in patients with diabetes mellitus. Int. J. Food Sci. Nutr..

[99] Oku T., Nakamura M., Takasugi A. (2009). Effects of cake made from whole soy powder on postprandial blood glucose and insulin levels in human subjects. Int. J. Food Sci. Nutr..

[100] Liu C., Lin X.L., Wan Z., Zou Y., Cheng F.F., Yang X.Q. (2016). The physicochemical properties, in vitro binding capacities and in vivo hypocholesterolemic activity of soluble dietary fiber extracted from soy hulls. Food Funct..

[101] Au M.M.C., Goff D., Kisch J.A., Coulson A., Wright A.J. (2013). Effects of soy-soluble fiber and flaxseed gum on the glycemic and insulinemic responses to glucose solutions and dairy products in healthy adult males. J. Am. Coll. Nutr..

[102] Tokede O.A., Onabanjo T.A., Yansane A., Gaziano J.M., Djoussé L. (2015). Soya products and serum lipids: A meta-analysis of randomised controlled trials. Br. J. Nutr..

[103] Marino M., Galluzzo P., Ascenzi P. (2006). Estrogen signaling multiple pathways to impact gene transcription. Curr. Genom..

[104] Ricketts M.-L., Moore D.D., Banz W.J., Mezei O., Shay N.F. (2005). Molecular mechanisms of action of the soy isoflavones includes activation of promiscuous nuclear receptors. A review. J. Nutr. Biochem..

[105] Engelbert A.K., Soukup S.T., Roth A., Hoffmann N., Graf D., Watzl B., Kulling S.E., Bub A. (2016). Isoflavone supplementation in postmenopausal women does not affect leukocyte LDL receptor and scavenger receptor CD36 expression: A double-blind, randomized, placebo-controlled trial. Mol. Nutr. Food Res..

[106] Turner R., Baron T., Wolffram S., Minihane A.M., Cassidy A., Rimbach G., Weinberg P.D. (2004). Effect of circulating forms of soy isoflavones on the oxidation of low density lipoprotein. Free Radic. Res..

[107] Xiao C.W., Wood C.M., Weber D., Aziz S.A., Mehta R., Griffin P., Cockell K.A. (2014). Dietary supplementation with soy isoflavones or replacement with soy proteins prevents hepatic lipid droplet accumulation and alters expression of genes involved in lipid metabolism in rats. Genes Nutr..

[108] Kobayashi M., Egusa S., Fukuda M. (2014). Isoflavone and protein constituents of lactic acid-fermented soy milk combine to prevent dyslipidemia in rats fed a high cholesterol diet. Nutrients.

[109] Yang B., Chen Y., Xu T.C., Yu Y.H., Huang T., Hu X.J., Li D. (2011). Systematic review and meta-analysis of soy products consumption in patients with type 2 diabetes mellitus. Asia Pac. J. Clin. Nutr..

[110] Huang H., Xie Z., Boue S.M., Bhatnagar D., Yokoyama W., Yu L., Wang T.T.Y. (2013). CZolesterol-lowering activity of soy-derived glyceollins in the golden Syrian hamster model. J. Agric. Food Chem..

[111] Nagata Y., Yamasaki S., Torisu N., Suzuki T., Shimamoto S., Tamaru S., Tanaka K. (2016). Okara, a by-product of tofu manufacturing, modifies triglyceride metabolism at the intestinal and hepatic levels. J. Nutr. Sci. Vitaminol..

[112] Sahebkar A. (2013). Fat lowers fat: Purified phospholipids as emerging therapies for dyslipidemia. Biochem. Biophys. Acta-Mol. Cell Biol. Lipids.

[113] Ovesen L., Ebbesen K., Olesen E.S. (1985). The Effects of oral soybean phospholipid on serum total cholesterol, plasma triglyceride, and serum high-density lipoprotein cholesterol concentrations in hyperlipidemia. J. Parent. Enter. Nutr..

[114] Spilburg F., Goldberg A.C., McGill J.B., Stenson W.F., Racette S.B., Bateman J., McPherson T.B., Ostlun R.E. (2003). Fat-free foods supplemented with soy stanol-lecithin powder reduce cholesterol absorption and LDL cholesterol. J. Am. Diet. Assoc..

[115] Høie L.H., Morgenstern E.C., Gruenwald J., Graubaum H.J., Busch R., Lüder W., Zunft H.J. (2005). A double-blind placebo-controlled clinical trial compares the cholesterol-lowering effects of two different soy protein preparations in hypercholesterolemic subjects. Eur. J. Nutr..

[116] Lukaczer D., Liska D.J., Lerman R.H., Darland G., Schilz B., Tripp M., Bland J.S. (2006). Effect of a low glycemic index diet with soy protein and phytosterols on CVD risk factors in postmenopausal women. Nutrition.

[117] Dong S., Zhang R., Ji Y., Hao J.Y., Ma W.W., Chen X.D., Xiao R., Yu H.L. (2016). Soy milk powder supplemented with phytosterol esters reduced serum cholesterol level in hypercholesterolemia independently of lipoprotein E genotype: A random clinical placebo-controlled trial. Nutr. Res..

[118] Butteiger D.N., Hibberd A.A., McGraw N.J., Napawan N., Hall-Porter J.M., Krul E.S. (2016). Soy protein compared with milk protein in a western diet increases gut microbial diversity and reduces serum lipids in golden Syrian hamsters. J. Nutr..

[119] Libby P. (2006). Inflammation and cardiovascular disease mechanisms. Am. J. Clin. Nutr..

[120] Han S., Wu H., Li W., Gao P. (2015). Protective effects of genistein in homocysteine-induced endothelial cell inflammatory injury. Mol. Cell. Biochem..

[121] Hernandez-Montes E., Pollard S.E., Vauzour D., Jofre-Montseny L., Rota C., Rimbach G., Weinberg P.D., Spencer J.P.E. (2006). Activation of glutathione peroxidase via Nrf1 mediates genistein’s protection against oxidative endothelial cell injury. Biochem. Biophys. Res. Commun..

[122] Sakamoto Y., Kanatsu J., Toh M., Naka A., Kondo K., Iida K. (2016). The dietary isoflavone daidzein reduces expression of pro-inflammatory genes through PPARα/γ and JNK pathways in adipocyte and macrophage co-cultures. PLoS ONE.

[123] Lesinski G.B., Reville P.K., Mace T.A., Young G.S., Ahn-Jarvis J., Thomas-Ahner J., Vodovotz Y., Ameen Z., Grainger E., Riedl K. (2015). Consumption of soy isoflavone enriched bread in men with prostate cancer is associated with reduced proinflammatory cytokines and immunosuppressive cells. Cancer Prev. Res..

[124] Ferguson J.F., Ryan M.F., Gibney E.R., Brennan L., Roche H.M., Reilly M.P. (2014). Dietary isoflavone intake is associated with evoked responses to inflammatory cardiometabolic stimuli and improved glucose homeostasis in healthy volunteers. Nutr. Metab. Cardiovasc. Dis..

[125] Llaneza P., González C., Fernandez-Iñarrea J., Alonso A., Diaz F., Arnott I., Ferrer-Barriendos J. (2011). Soy isoflavones, diet and physical exercise modify serum cytokines in healthy obese postmenopausal women. Phytomedicine.

[126] Azadbakht L., Atabak S., Esmaillzadeh A. (2008). Soy protein intake, cardiorenal indices, and C-reactive protein in type 2 diabetes. Diabetes Care.

[127] Tomayko E.J., Kistler B.M., Fitschen P.J., Wilund K.R. (2015). Intradialytic protein supplementation reduces inflammation and improves physical function in maintenance hemodialysis patients. J. Ren. Nutr..

[128] Reverri E.J., LaSalle C.D., Franke A.A., Steinberg F.M. (2015). Soy provides modest benefits on endothelial function without affecting inflammatory biomarkers in adults at cardiometabolic risk. Mol. Nutr. Food Res..

[129] Nakamura H., Takasawa M., Kashara S., Tsuda A., Momotsu T., Ito S., Shibata A. (1989). Effects of acute protein loads of different sources on renal function of patients with diabetic nephropathy. Tohoku J. Exp. Med..

[130] Mauriz J.L., Matilla B., Culebras J.M., Gonzalez P., Gonzalez-Gallego J. (2001). Dietary glycine inhibits activation of nuclear factor kappa B and prevents liver injury in hemorrhagic shock in the rat. Free Radic. Biol. Med..

[131] DeBoer M.D. (2013). Obesity, systemic inflammation, and increased risk for cardiovascular disease and diabetes among adolescents: A need for screening tools to target interventions. Nutrition.

[132] Furukawa S., Fujita T., Shimabukuro M., Iwaki M., Yamada Y., Nakajima Y., Nakayama O., Makishima M. (2004). Increased oxidative stress in obesity and its impact on metabolic syndrome. J. Clin. Investig..

[133] Chaiswing L., Cole M.P., St. Clair D.K., Ittarat W., Szweda L.I., Oberley T.D. (2004). Oxidative damage precedes nitrative damage in adriamycin-induced cardiac mitochondrial injury. Toxicol. Pathol..

[134] Kurrat A., Blei T., Kluxen F.M., Mueller D.R., Piechotta M., Soukup S.T., Kulling S.E., Diel P. (2015). Lifelong exposure to dietary isoflavones reduces risk of obesity in ovariectomized Wistar rats. Mol. Nutr. Food Res..

[135] Huang C., Pang D., Luo Q., Chen X., Gao Q., Shi L., Liu W., Zou Y., Li L., Chen Z. (2016). Soy Isoflavones regulate lipid metabolism through an AKT/mTORC1 pathway in diet-induced obesity (DIO) male rats. Molecules.

[136] Yang H., Li F., Xiong X., Kong X., Zhang B., Yuan X., Fan J., Duan Y., Geng M., Li L. (2013). Soy isoflavones modulate adipokines and myokines to regulate lipid metabolism in adipose tissue, skeletal muscle and liver of male Huanjiang mini-pigs. Mol. Cell. Endocrinol..

[137] Rupasinghe H.P.V., Sekhon-Loodu S., Mantso T., Panayiotidis M.I. (2016). Phytochemicals in regulating fatty acid β-oxidation: Potential underlying mechanisms and their involvement in obesity and weight loss. Pharmacol. Ther..

[138] Zanella I., Marrazzo E., Biasiotto G., Penza M., Romani A., Vignolini P., Caimi L., Di Lorenzo D. (2015). Soy and the soy isoflavone genistein promote adipose tissue development in male mice on a low-fat diet. Eur. J. Nutr..

[139] Giordano E., Dávalos A., Crespo M.C., Tomé-Carneiro J., Gómez-Coronado D., ViZioli F. (2015). Soy isoflavones in nutritionally relevant amounts have varied nutrigenomic effects on adipose tissue. Molecules.

[140] Oh H.-G., Kang Y.-R., Lee H.-Y., Kim J.-H., Shin E.-H., Lee B.-G., Park S.-H., Moon D.-I., Kim O.-J., Lee I.-A. (2014). Ameliorative effects of Monascus pilosus-fermented black soybean (*Glycine max* L. Merrill) on high-fat diet-induced obesity. J. Med. Food.

[141] Wu J., Oka J., Tabata I., Higuchi M., Toda T., Fuku N., Ezaki J., Sugiyama F., Uchiyama S., Yamada K. (2006). Effects of isoflavone and exercise on BMD and fat mass in postmenopausal Japanese women: A 1-year randomized placebo-controlled trial. J. Bone Miner. Res..

[142] Matvienko O.A., Alekel D.L., Genschel U., Ritland L., Van Loan M.D., Koehler K.J. (2010). Appetitive hormones, but not isoflavone tablets, influence overall and central adiposity in healthy postmenopausal women. Menopause.

[143] Frankenfeld C.L., Atkinson C., Wähälä K., Lampe J.W. (2014). Obesity prevalence in relation to gut microbial environments capable of producing equol or *O*-desmethylangolensin from the isoflavone daidzein. Eur. J. Clin. Nutr..

[144] Kani A.H., Alavian S.M., Esmaillzadeh A., Adibi P., Azadbakht L. (2014). Effects of a novel therapeutic diet on liver enzymes and coagulating factors in patients with non-alcoholic fatty liver disease: A parallel randomized trial. Nutrition.

[145] Suga Y., Tanabe K., Kim J., Sato H., Iwashita S., Hamada K., Kuno S. (2013). A comparison of the influences of soy- vs. wheat-based supplements on weight loss in middle-aged subjects. Int. J. Sport Health Sci..

[146] Beavers K.M., Gordon M.M., Easter L., Beavers D.P., Hairston K.G., Nicklas B.J., Vitolins M.Z. (2015). Effect of protein source during weight loss on body composition, cardiometabolic risk and physical performance in abdominally obese, older adults: A pilot feeding study. J. Nutr. Health Aging.

[147] Takahira M., Noda K., Fukushima M., Zhang B., Mitsutake R., Uehara Y., Ogawa M., Kakuma T., Saku K. (2011). Randomized, double-blind, controlled, comparative trial of formula food containing soy protein vs. milk protein in visceral fat obesity. Circ. J..

[148] Kok L., Kreijkamp-Kaspers S., Grobbee D.E., Lampe J.W., van der Schouw Y.T. (2005). Soy isoflavones, body composition, and physical performance. Maturitas.

[149] Kwak J.H., Ahn C.W., Park S.H., Jung S.U., Min B.J., Kim O.Y., Lee J.H. (2012). Weight reduction effects of a black soy peptide supplement in overweight and obese subjects: Double blind, randomized, controlled study. Food Funct..

[150] Koohkan S., Schaffner D., Milliron B.J., Frey I., König D., Deibert P., Vitolins M., Berg A. (2014). The impact of a weight reduction program with and without meal-replacement on health related quality of life in middle-aged obese females. BMC Womens Health.

[151] Weickert M.O., Reimann M., Otto B., Hall W.L., Vafeiadou K., Hallund J., Ferrari M., Talbot D., Branca F., Bügel S. (2006). Soy isoflavones increase preprandial peptide YY (PYY), but have no effect on ghrelin and body weight in healthy postmenopausal women. J. Negat. Results Biomed..

[152] Simmons A.L., Miller C.K., Clinton S.K., Vodovot Y. (2011). A comparison of satiety, glycemic index, and insulinemic index of wheat-derived soft pretzels with or without soy. Food Funct..

[153] Douglas S.M., Lasley T.R., Leidy H.J. (2015). Consuming beef vs. soy protein has little effect on appetite, satiety, and food intake in healthy Adults. J. Nutr..

[154] Neacsu M., Fyfe C., Horgan G., Johnstone A.M. (2014). Appetite control and biomarkers of satiety with vegetarian (soy) and meat-based high-protein diets for weight loss in obese men: A randomized crossover trial. Am. J. Clin. Nutr..

[155] Tahavorgar A., Vafa M., Shidfar F., Gohari M., Heydari I. (2014). Whey protein preloads are more beneficial than soy protein preloads in regulating appetite, calorie intake, anthropometry, and body composition of overweight and obese men. Nutr. Res..

[156] Padhi E.M.T., Ramdath D.D., Carson S.J., Hawke A., Blewett H.J., Wolever T.M.S., Vella D., Seetharaman K., Duizer L.M., Duncan A.M. (2015). Liking of soy flour muffins over time and the impact of a health claim on willingness to consume. Food Res. Int..

[157] Leidy H.J., Todd C.B., Zino A.Z., Immel J.E., Mukherjea R., Shafer R.S., Ortinau L.C., Braun M. (2015). Consuming high-protein soy snacks affects appetite control, satiety, and diet quality in young people and influences select aspects of mood and cognition. J. Nutr..

[158] König D., Muser K., Berg A., Deibert P. (2012). Fuel selection and appetite-regulating hormones after intake of a soy protein-based meal replacement. Nutrition.

[159] Huang X.F., Liu H., Rahardjo G.L., Mclennan P.L., Tapsell L.C., Buttemer W.A. (2008). Effects of diets high in whey, soy, red meat and milk protein on body weight maintenance in diet-induced obesity in mice. Nutr. Diet..

